# Development and validation of a reinforcement learning model for ventilation control during emergence from general anesthesia

**DOI:** 10.1038/s41746-023-00893-w

**Published:** 2023-08-14

**Authors:** Hyeonhoon Lee, Hyun-Kyu Yoon, Jaewon Kim, Ji Soo Park, Chang-Hoon Koo, Dongwook Won, Hyung-Chul Lee

**Affiliations:** 1 Department of Anesthesiology and Pain Medicine, Seoul National University Hospital, Seoul, Republic of Korea; 2 Biomedical Research Institute, Seoul National University Hospital, Seoul, Republic of Korea; 3 Department of Anesthesiology and Pain Medicine, Seoul National University College of Medicine, Seoul National University Hospital, Seoul, Republic of Korea; 4 Center for Digital Health, Medical Science Research Institute, Kyung Hee University Medical Center, Kyung Hee University College of Medicine, Seoul, Republic of Korea; 5 Department of Pediatrics, Seoul National University College of Medicine, Seoul National University Hospital, Seoul, Republic of Korea; 6 Department of Anesthesiology and Pain Medicine, Seoul National University Bundang Hospital, Seongnam, Republic of Korea; 7 Department of Anesthesiology and Pain Medicine, SMG-SNU Boramae Medical Center, Seoul National University College of Medicine, Seoul, Republic of Korea

**Keywords:** Risk factors, Outcomes research

## Abstract

Ventilation should be assisted without asynchrony or cardiorespiratory instability during anesthesia emergence until sufficient spontaneous ventilation is recovered. In this multicenter cohort study, we develop and validate a reinforcement learning-based Artificial Intelligence model for Ventilation control during Emergence (AIVE) from general anesthesia. Ventilatory and hemodynamic parameters from 14,306 surgical cases at an academic hospital between 2016 and 2019 are used for training and internal testing of the model. The model’s performance is also evaluated on the external validation cohort, which includes 406 cases from another academic hospital in 2022. The estimated reward of the model’s policy is higher than that of the clinicians’ policy in the internal (0.185, the 95% lower bound for best AIVE policy vs. −0.406, the 95% upper bound for clinicians’ policy) and external validation (0.506, the 95% lower bound for best AIVE policy vs. 0.154, the 95% upper bound for clinicians’ policy). Cardiorespiratory instability is minimized as the clinicians’ ventilation matches the model’s ventilation. Regarding feature importance, airway pressure is the most critical factor for ventilation control. In conclusion, the AIVE model achieves higher estimated rewards with fewer complications than clinicians’ ventilation control policy during anesthesia emergence.

## Introduction

The emergence from general anesthesia is dynamic, and various physiologic responses can occur during this phase^1^. Restoration of spontaneous breathing is one of the first physiological signs that appear during the emergence from general anesthesia^2^. When the patient’s spontaneous breathing begins to recover, anesthesiologists switch off the mechanical ventilator and assist the patient with manual ventilation at optimal timing to avoid complications, such as cardiorespiratory instability or patient-ventilator asynchrony.

Since most of the ventilation control during emergence is performed by human clinicians, human factors can affect the risk of emergence from anesthesia^3^. Especially, anesthesiologists can be unassisted at the end of the surgery and distracted by the high task load and fatigue. Generally, the situation during emergence from anesthesia is less controlled than at induction.

Artificial intelligence algorithms can assist human clinicians in various medical fields^4,5^. Among the artificial intelligence algorithms, the reinforcement learning algorithms can find the optimal policy by maximizing the cumulative expected reward^6^. This is similar to the decision-making process of a clinician whose goal is improving the clinical outcome through appropriate intervention^7^. In previous studies, reinforcement learning algorithms have been used for various medical problems^8^, such as drug administration during general anesthesia^9^, hypotension treatment^10^, and ventilation settings in the intensive care unit^11^.

In this study, we aim to develop and validate the reinforcement learning-based Artificial Intelligence model for Ventilation during Emergence (AIVE) from general anesthesia to control ventilation during emergence from general anesthesia while preventing hemodynamic and ventilatory complications. We hypothesize that compared to the clinicians’ policy, AIVE’s policy would achieve higher estimated rewards defined by the clinical outcomes.

## Results

### Dataset construction

**Fig. 1:**
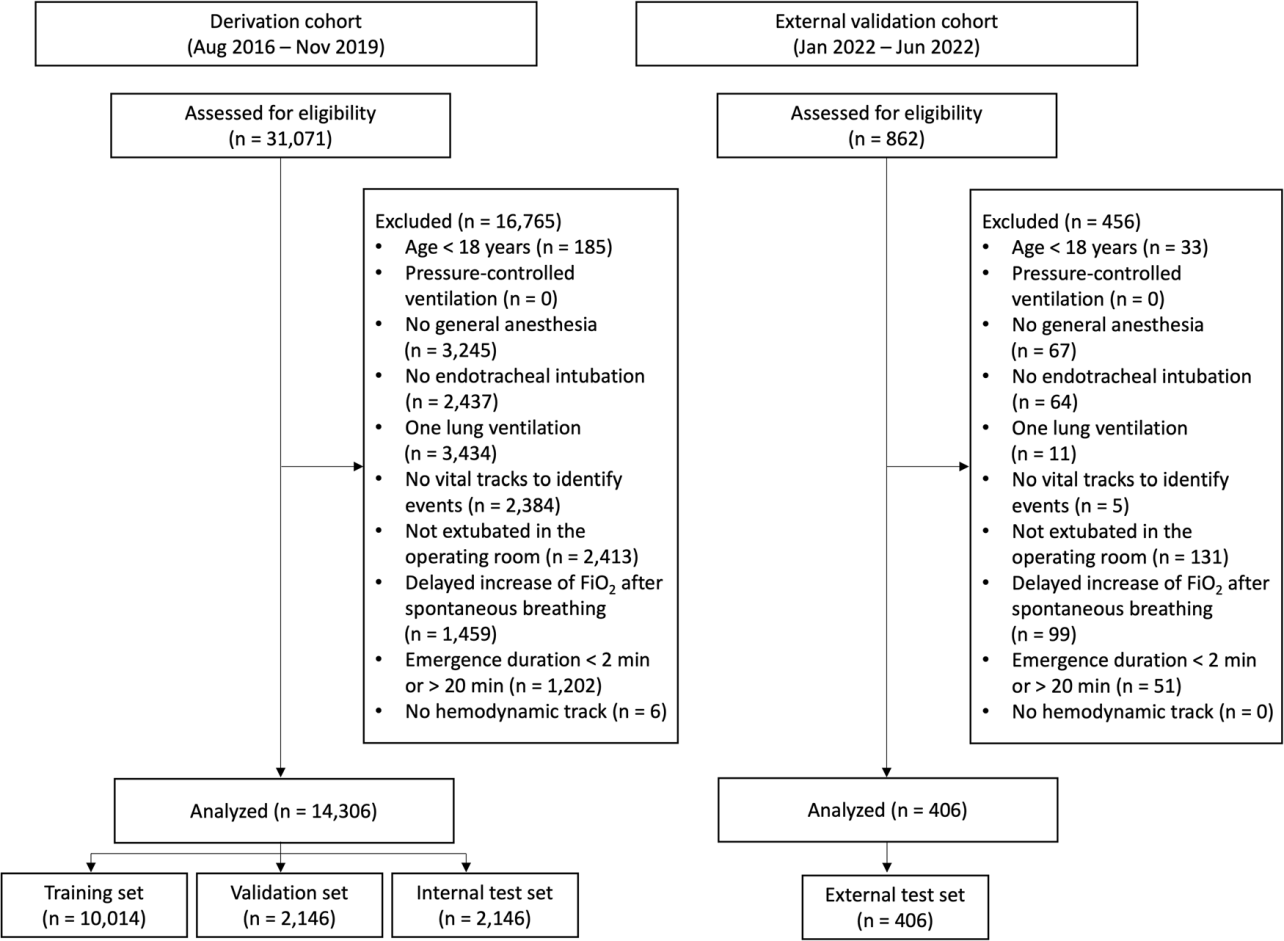
Flow chart of dataset construction. FiO_2_ a fraction of inspired oxygen.

Among the 31,071 cases from the derivation cohort, 14,306 cases (6,763,535 one-second time points) were included for model development and internal validation (Fig. 1). From the derivation cohort, 2146 cases (15%) were randomly selected for internal validation. The remaining cases (85%) were used for model training and hyperparameter tuning. External validation was performed using 406 cases (162,656 one-second time points) from the independent dataset from the external validation cohort. The demographic data and perioperative features of the analyzed cases are presented in Table 1.

**Table 1:** Demographic, anesthetic, and surgical characteristics of the study population.

	Derivation cohort (*n* = 14,306)	External validation cohort (*n* = 406)
Age (year)	57.8 ± 14.8	54.5 ± 16.0
Sex		
Female	7225 (50.5%)	223 (54.9%)
Male	7081 (49.5%)	183 (45.1%)
Height (cm)	162.6 ± 9.1	NA
Weight (kg)	64.0 ± 12.2	NA
The total duration of anesthesia (min)	164.5 ± 12.8	134.5 ± 81.5
Duration of anesthesia emergence (s)	469 ± 210	407 ± 199
Surgery type, (%)		
General surgery	8917 (62.3%)	346 (85.2%)
Urologic surgery	1909 (13.3%)	1 (0.2%)
Orthopedic surgery	1201 (8.4%)	12 (3.0%)
Gynecological surgery	790 (5.5%)	40 (9.9%)
Neurosurgery	644 (4.5%)	6 (1.5%)
Plastic surgery	614 (4.3%)	0 (0.0%)
Thoracic surgery	97 (0.7%)	0 (0.0%)
Others	134 (0.9%)	1 (0.2%)

Data are presented as mean ± standard deviation or number (%). *NA* not available.

### Performance evaluation

**Fig. 2:**
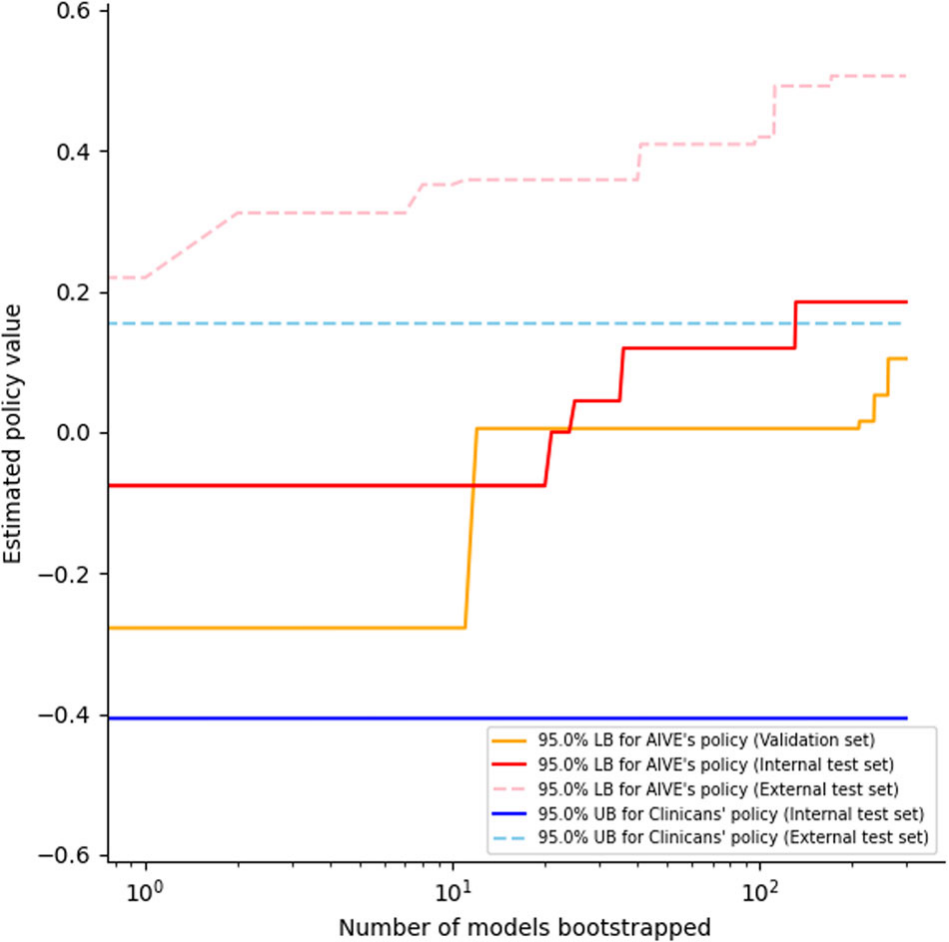
Performances of the AIVE’s and clinicians’ policies. The estimated policy values are derived from the normalized rewards (from −1 to 1), indicating that 0 is the mean policy value of the derivation cohort. LB lower bound, AIVE Artificial Intelligence model for Ventilation control during Emergence, UB upper bound.

Three hundred models were built from the training set to compare the estimated rewards of AIVE’s policy with those of the clinicians’ policy. The whole learning scheme was consistent for each model. The model’s estimated rewards were significantly higher than the clinician’s rewards in the internal validation (0.185, the 95% lower bound for best AIVE policy vs. −0.406, the 95% upper bound for clinicians’ policy) and the testing set (0.506, the 95% lower bound for best AIVE policy vs. 0.154, the 95% upper bound for clinicians’ policy). As shown in Fig. 2, the 95% lower bound of the estimated rewards of the AIVE’s policy consistently exceeded the 95% upper bound of the estimated performance return of the clinicians’ policy in the internal validation and external validation sets, suggesting that a sufficient number of models were developed.

**Fig. 3:**
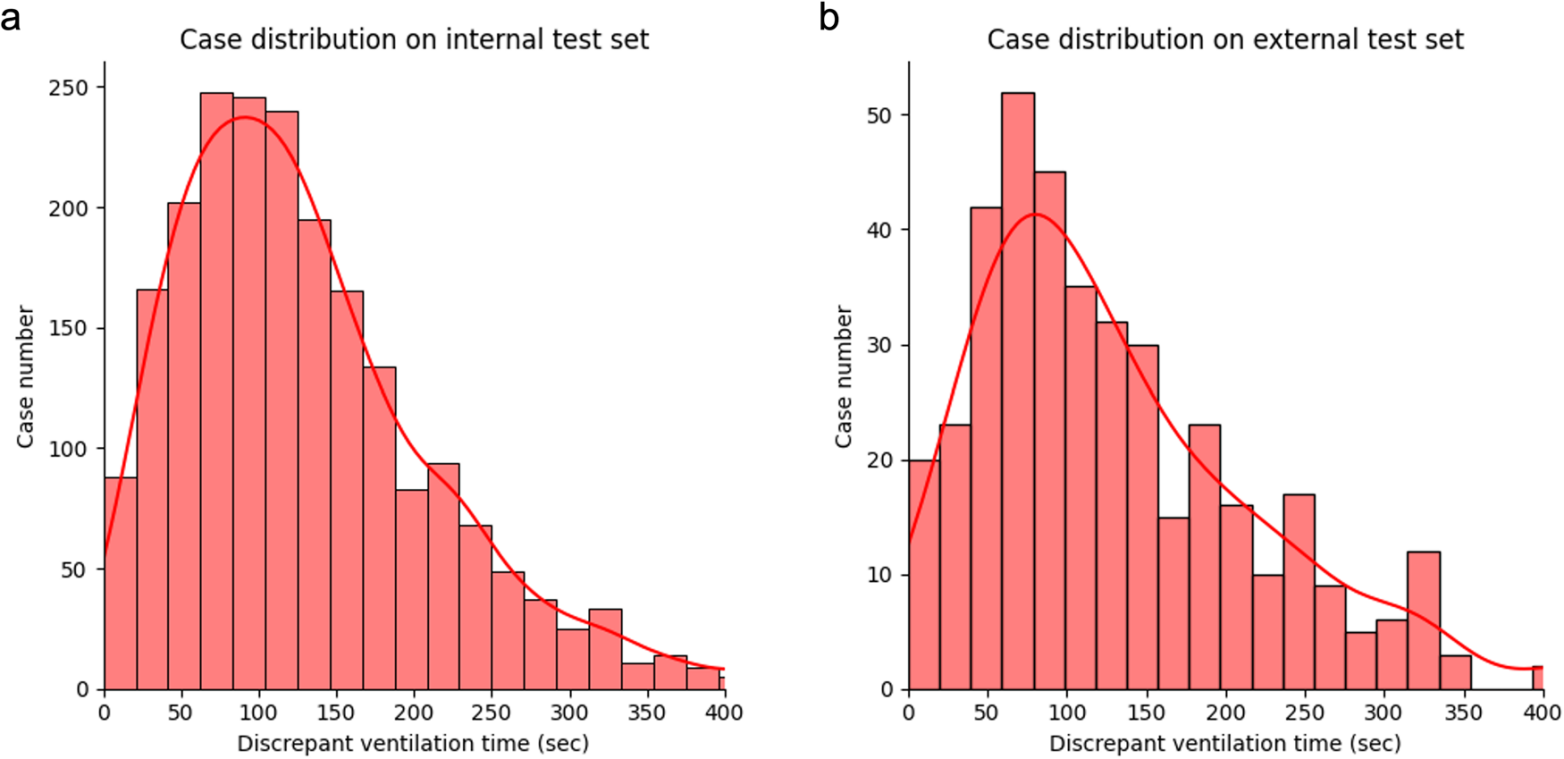
The distribution of discrepancy between the AIVE’s and clinicians’ policies. **a** The distribution of discrepancy between the two policies in the internal test set. **b** The discrepancy distribution between the two policies in the external test set. AIVE Artificial Intelligence model for Ventilation control during Emergence.

The distribution of discrepancy between the AIVE’s and clinicians’ policies is presented in Fig. 3. In most cases, the time discrepancy between the two policies for suggesting ventilation was within 2 min in the internal validation set and a minute in the external validation set, indicating that the AIVE’s policy could be developed from the suboptimal clinicians’ policy.

### Outcome differences

**Fig. 4:**
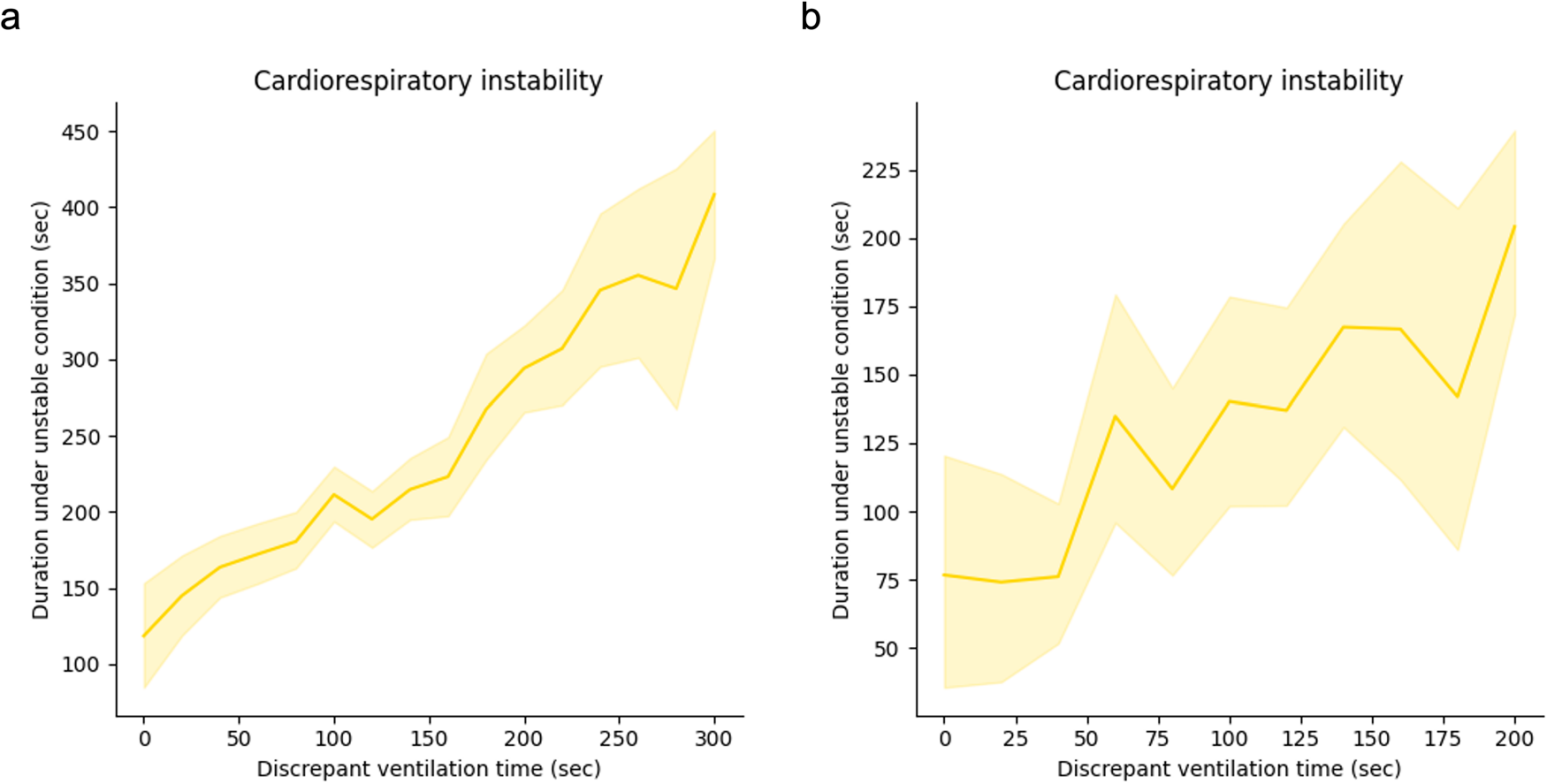
The changes in cardiorespiratory instability depend on the degree of time discrepancy between the AIVE’s and clinicians’ policies. The plots were produced with 3000 resamplings, and the shaded area represents the 95% confidence interval. **a** The cardiorespiratory instability changes depending on the time discrepancy between the two policies in the internal test set. **b** The cardiorespiratory instability changes depending on the time discrepancy between the two policies in the external test set. AIVE Artificial Intelligence model for Ventilation control during Emergence.

**Fig. 5:**
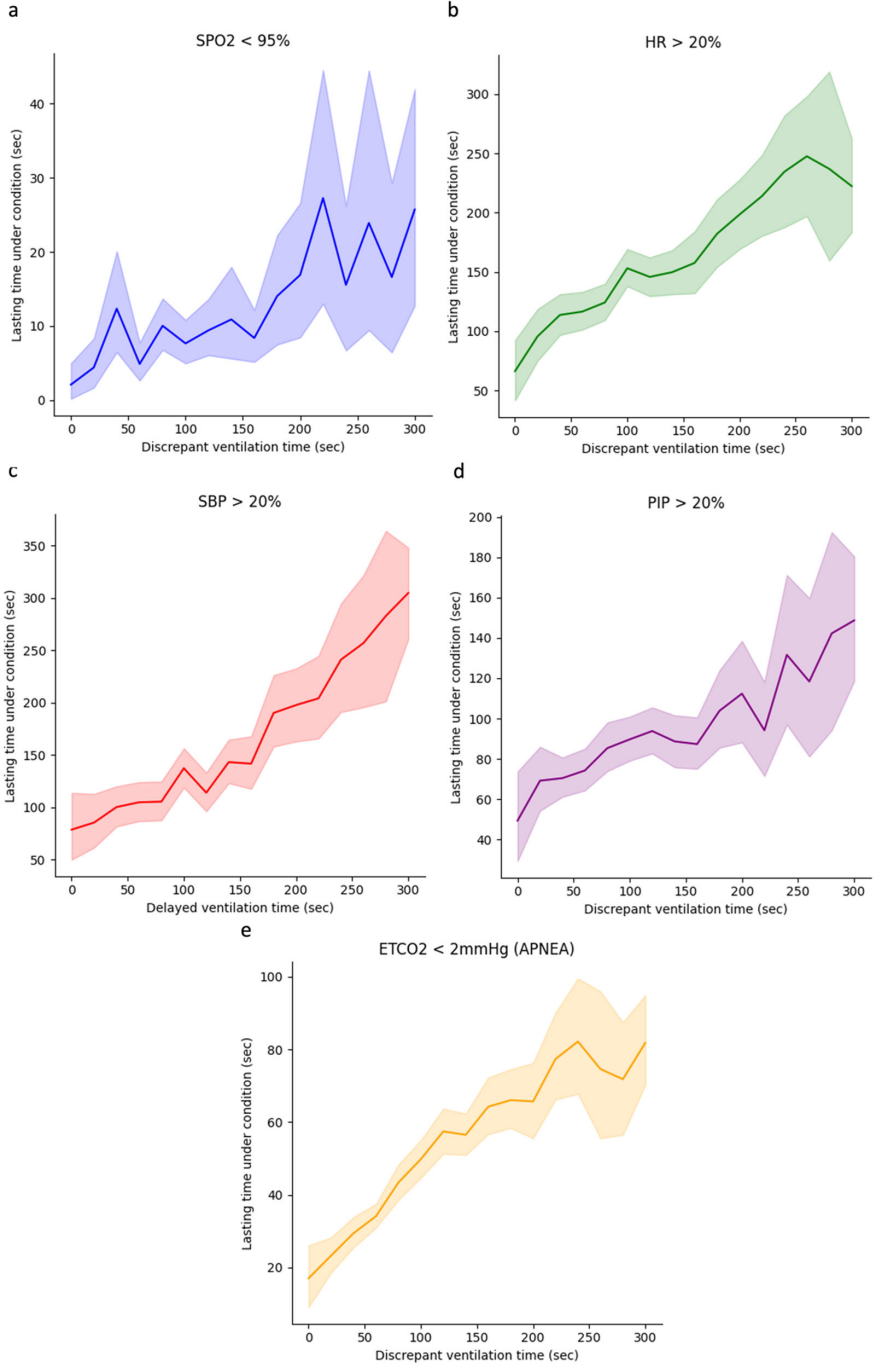
The changes in cardiorespiratory parameters depend on the degree of time discrepancy between the AIVE’s and clinicians’ policies in the internal test set. The plots were produced with 3,000 resamplings, and the shaded area represents the 95% confidence interval. **a** SpO_2_ <95%, **b** HR >20%, **c** SBP >20%, **d** PIP >20%. **e** E_T_CO_2_ <2 mmHg. SpO_2_ peripheral oxygen saturation, HR heart rate, SBP systolic blood pressure, PIP peak inspiratory pressure, E_T_CO_2_ end-tidal carbon dioxide concentration, AIVE Artificial Intelligence model for Ventilation control during Emergence.

**Fig. 6:**
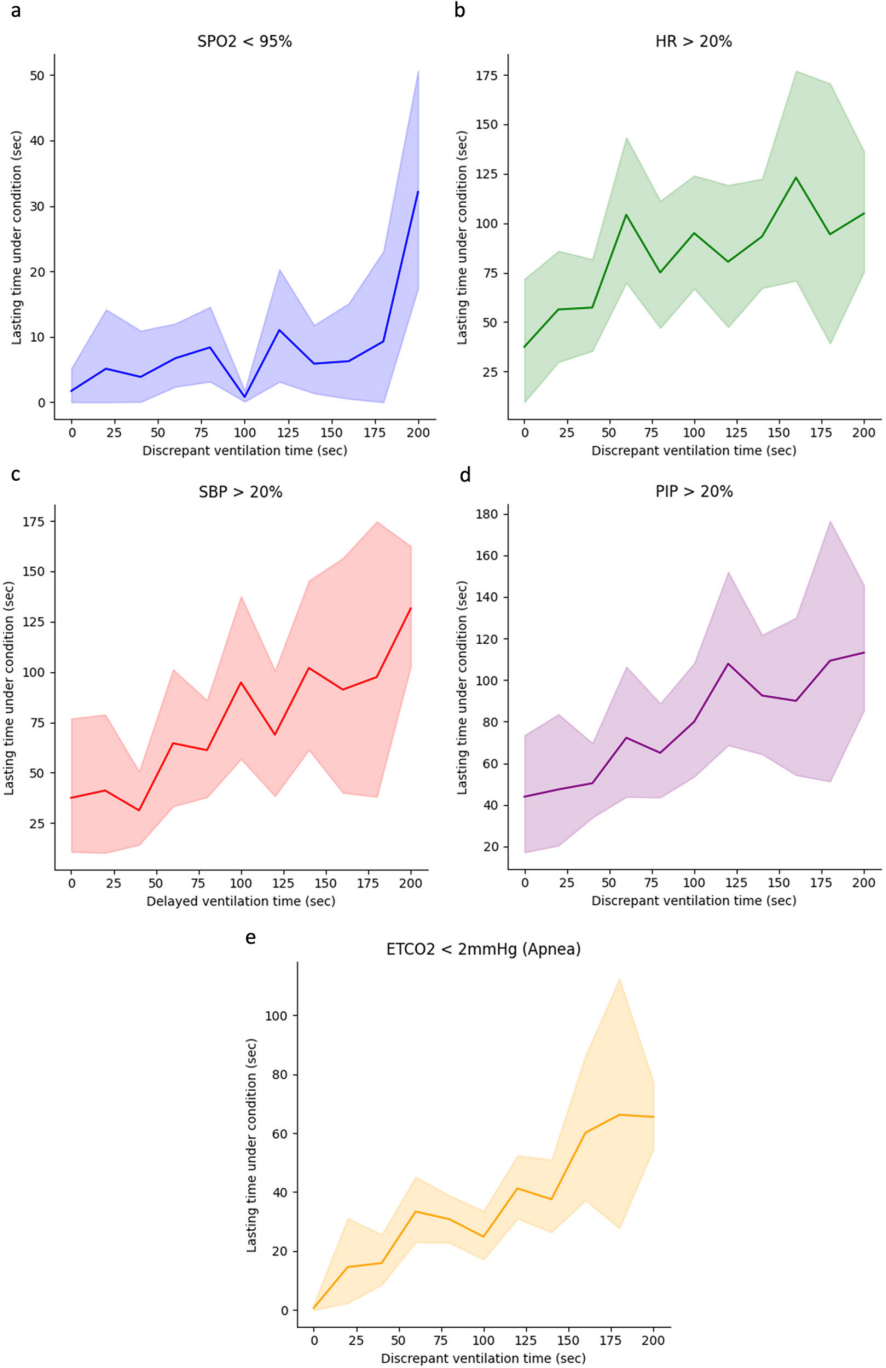
The changes in cardiorespiratory parameters depend on the degree of time discrepancy between the AIVE’s and clinicians’ policies in the external test dataset. The plots were produced with 3000 resamplings, and the shaded area represents the 95% confidence interval. **a** SpO_2_ <95%, **b** HR >20%, **c** SBP >20%, **d** PIP >20%, **e** E_T_CO_2_ <2 mmHg. SpO_2_ peripheral oxygen saturation, HR heart rate, SBP systolic blood pressure, PIP peak inspiratory pressure, E_T_CO_2_ end-tidal carbon dioxide concentration, AIVE Artificial Intelligence model for Ventilation control during Emergence.

Mismatched ventilation by a clinician’s policy during the emergence process with the AIVE’s policy was associated with increasing cardiorespiratory instability in a time-dependent manner (Fig. 4 and Table 2). There was a significant positive correlation between mismatched ventilation and increased cardiorespiratory instability in the internal and external test sets (All *P* < 0.001). As the secondary outcomes, the correlation between the cardiorespiratory parameters, including peripheral oxygen saturation (SpO_2_), heart rate (HR), systolic blood pressure (SBP), peak inspiratory pressure (PIP), and end-tidal carbon dioxide concentration (E_T_CO_2_) in the internal and external test sets, are presented in Figs. 5 and 6, Table 2, and Supplementary Figs. 1 and 2. Significant positive correlations were observed between all cardiorespiratory parameters and the time of policy discrepancy in the internal test set, which was the same as the primary outcome. In the external test set, most cardiorespiratory parameters, except for HR, showed significant positive correlations with the time of policy discrepancy. Regarding secondary outcomes, significant positive correlations were observed between the policy discrepancy and the clinical outcomes (length of hospital stay and length of post-anesthesia care unit [PACU] stay) as well as cardiorespiratory parameters within 48 h after surgery (HR and respiratory rate [RR]). However, the length of hospital stay in the external test set was not statistically significant. Notably, SpO_2_ showed a significant negative correlation in the internal test set, suggesting that policy discrepancy could potentially lead to lower SpO_2_ levels after surgery. Among the patients who underwent chest X-rays (52.3% of the total) within 48 h after surgery, no significant correlations were found between the policy discrepancy and the incidence of atelectasis and pulmonary edema based on the X-ray results (Table 2). In addition, there were no significant correlations among the patients who had the arterial blood gas analysis after surgery (20.0% of the total). The results of subgroup analyses regarding the primary and secondary outcomes based on age, sex, and type of surgery are presented in Table 3 and Supplementary Tables 2 and 3. In all age and sex subgroups, substantial positive correlations were observed between the policy discrepancy and the cardiorespiratory instability in the internal test set. Regarding the surgical type, considerable positive correlations were detected between the policy discrepancy and cardiorespiratory instability among patients undergoing general, urological, orthopedic, gynecological, and neurosurgery.

**Table 2:** The correlation between the policy discrepancy and the primary and secondary outcomes in the internal and external test set.

	Internal test set			External test set		
	*N*	Coefficient	*P*-value	*N*	Coefficient	*P*-value
Primary outcome						
Cardiorespiratory instability	2146	0.252	<0.001	406	0.216	<0.001
Secondary outcomes						
Intraoperative cardiorespiratory parameters						
SpO_2_ <95%	2146	0.091	<0.001	406	0.143	<0.001
HR >20%	2146	0.155	<0.001	406	0.051	0.139
SBP >20%	2146	0.193	<0.001	406	0.194	<0.001
PIP >20%	2146	0.079	<0.001	406	0.114	<0.001
E_T_CO_2_ <2 mmHg	2146	0.275	<0.001	406	0.295	<0.001
Postoperative clinical outcomes						
Length of hospital stay	2146	0.099	<0.001	396	0.013	0.719
Length of PACU stay	2146	0.059	<0.001	NA		
30-day in-hospital death	2146	0.037	0.035	NA		
Postoperative outcomes within 48 h after surgery						
Chest X-ray						
Atelectasis	1122	−0.001	0.978	155	−0.073	0.271
Pulmonary edema	1122	0.039	0.114	155	−0.087	0.178
Cardiorespiratory profiles						
SpO_2_	1789	−0.049	0.002	NA		
HR	1853	0.065	<0.001	NA		
SBP	1847	0.042	0.007	NA		
RR	1849	0.049	0.002	NA		
Arterial blood gas analysis						
PaO_2_	429	0.007	0.826	15	−0.150	0.021
PaCO_2_	430	−0.034	0.288	16	0.448	0.450

Kendall’s rank correlation analysis was performed for continuous outcomes, and the point-biserial correlation analysis for categorical outcomes.

*SpO*_*2*_ peripheral oxygen saturation, *HR* heart rate, *SBP* systolic blood pressure, *PIP* peak inspiratory pressure, *E*_*T*_*CO*_*2*_ end-tidal carbon dioxide concentration, *PACU* post-anesthesia care unit, *RR* respiratory rate; *PaO*_*2*_ partial pressure of oxygen, *PaCO*_*2*_ partial pressure of carbon dioxide, *NA* not available.

**Table 3:** Subgroup analysis for the cardiorespiratory instability in the internal and external test set.

	Internal test set			External test set		
	*N*	Coefficient	*P*-value	*N*	Coefficient	*P*-value
Age						
>50	1481	0.257	<0.001	247	0.267	<0.001
≤50	665	0.226	<0.001	159	0.112	0.038
Sex						
Female	1091	0.260	<0.001	223	0.297	<0.001
Male	1055	0.252	<0.001	183	0.131	0.009
Surgery type						
General surgery	1378	0.261	<0.001	346	0.213	<0.001
Urology surgery	239	0.215	<0.001	1	NA	
Orthopedic surgery	161	0.340	<0.001	12	0.076	0.731
Gynecological surgery	114	0.198	0.002	40	0.212	0.054
Neurosurgery	93	0.231	0.001	6	0.414	0.251
Plastic surgery	92	0.175	0.014	0	NA	
Thoracic surgery	19	0.129	0.441	0	NA	
Others	20	0.460	0.005	1	NA	

Kendall’s rank correlation analysis was performed. *NA* not available.

### Visualization of representative cases for comparison of policies

**Fig. 7:**
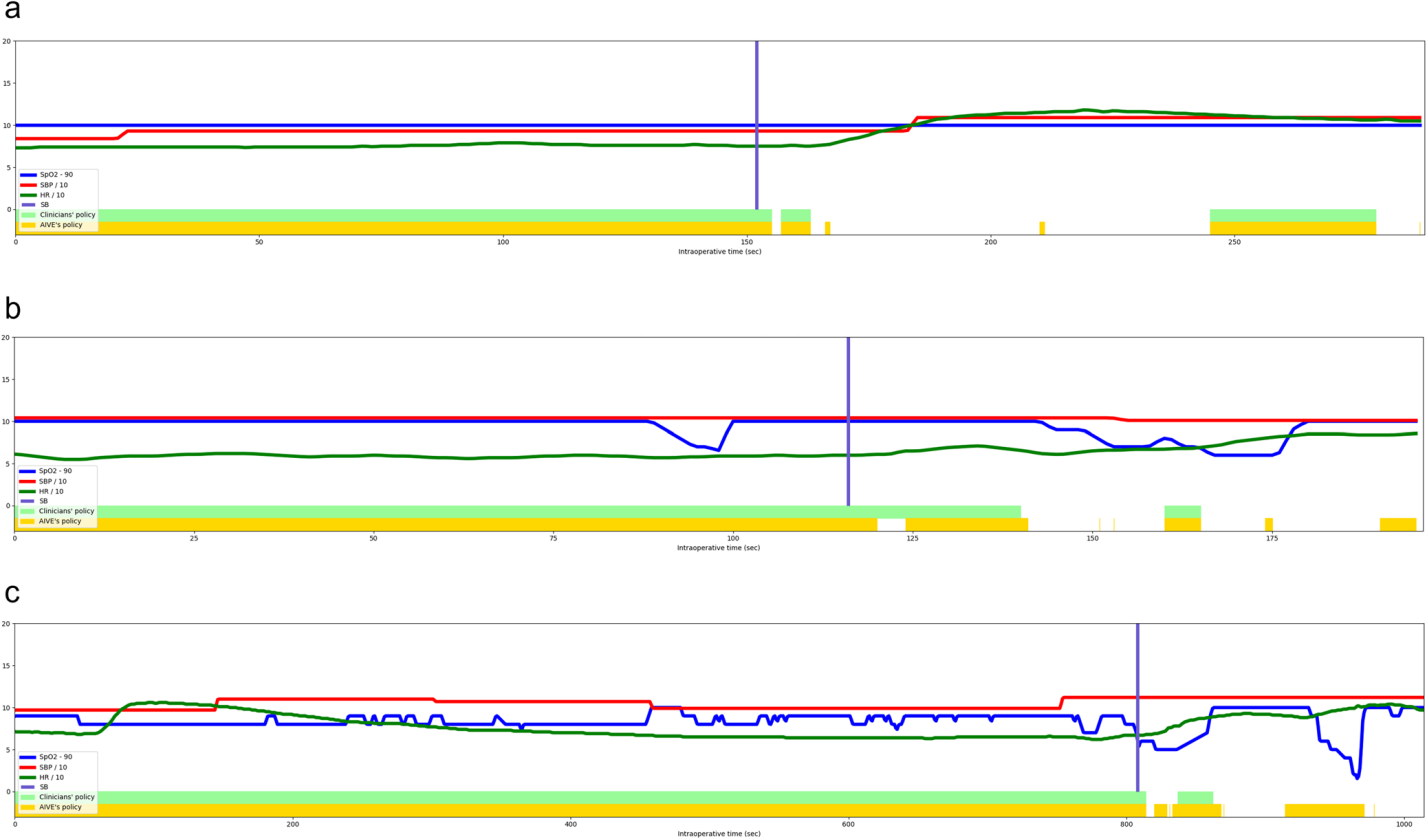
Three representative cases. **a** A case was maintaining a stable cardiorespiratory status during emergence from general anesthesia. **b** and **c** Two cases represent cardiorespiratory instability during emergence from general anesthesia. AIVE suggested turning the mechanical ventilator off earlier (**b**) and applying manual ventilation before developing cardiorespiratory deterioration (**b** and **c**). SpO_2_ peripheral oxygen saturation, HR heart rate, SBP systolic blood pressure, SB spontaneous breathing, AIVE Artificial Intelligence model for Ventilation control during Emergence.

Figure 7 shows two representative cases to identify the change in cardiorespiratory parameters with the discrepancy between AIVE’s and the clinician’s policies. Cardiorespiratory parameters are maintained during the emergence from general anesthesia when each policy’s ventilation control is consistent with the AIVE’s policy. However, cardiorespiratory parameters worsened when the clinicians’ actual control was discrepant with the AIVE’s policy. AIVE suggested controlling mechanical or manual ventilation based on the patient’s status to prevent excessive changes in the cardiorespiratory parameters.

### Feature importance

**Fig. 8:**
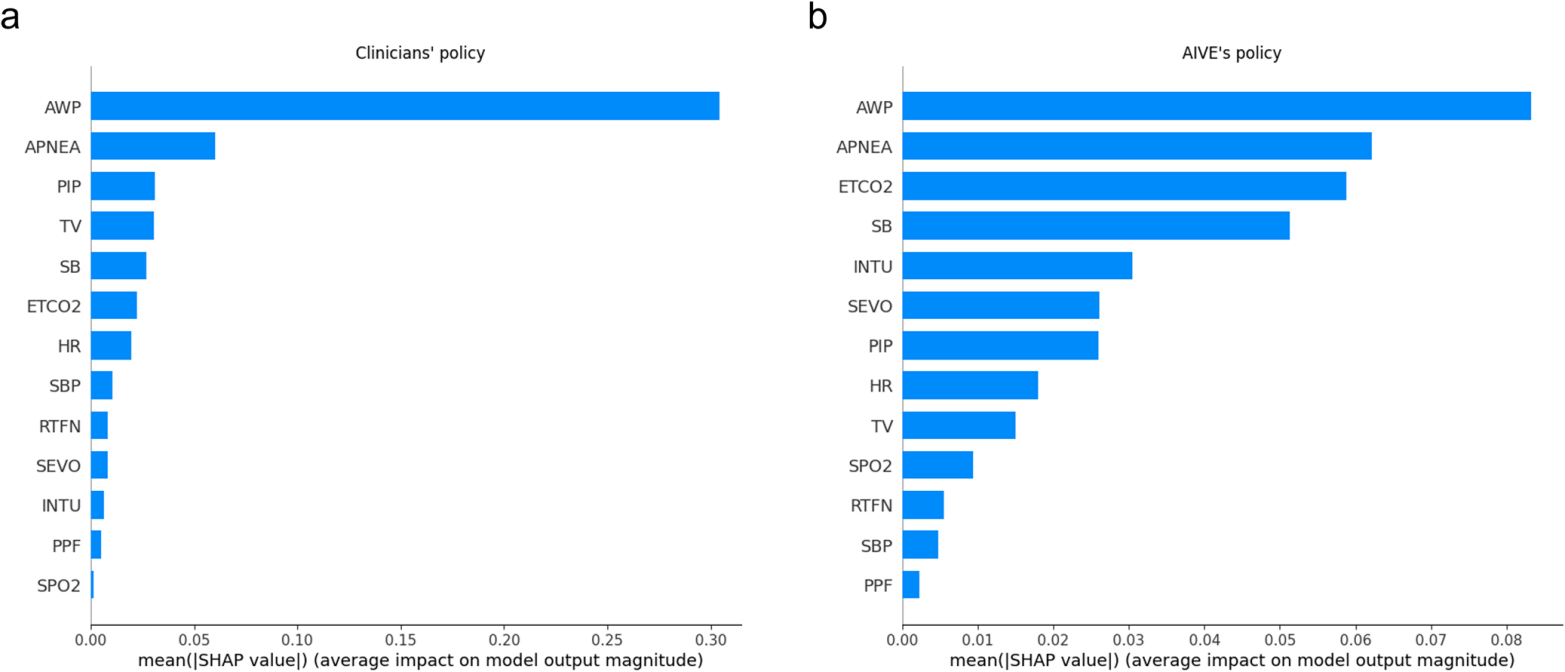
Feature importance from the SHAP method. **a** Feature importance of the AIVE’s policy. **b** Feature the importance of the clinicians’ policy. AWP airway pressure, PIP peak inspiratory pressure, TV tidal volume, SB spontaneous breathing, E_T_CO_2_ end-tidal carbon dioxide concentration, HR heart rate, SBP systolic blood pressure, RFTN remifentanil, SEVO sevoflurane, INTU intubation, PPF propofol, SpO_2_ peripheral oxygen saturation, AIVE Artificial Intelligence model for Ventilation control during Emergence.

The SHapley Additive exPlanations (SHAP) method was used to present the degree of importance of each feature for the AIVE’s and clinicians’ policies, respectively. The most important feature for controlling ventilation in both policies was the decreased airway pressure (AWP) (Fig. 8). However, unlike clinicians who usually focused only on the level of AWP, AIVE comprehensively considered other parameters, such as cumulative apnea time and spontaneous breathing.

## Discussion

The present study developed and externally validated a reinforcement learning model that controls ventilation during the emergence from general anesthesia. AIVE’s policy showed higher estimated rewards than the clinicians’ policy, indicating that the actions suggested by AIVE could be superior to those suggested by clinicians for maintaining cardiorespiratory stability, adequate oxygenation, and decarboxylation during the emergence from general anesthesia. As the discrepancy increased between the AIVE’s and clinicians’ ventilation, worse outcomes were observed.

The advantages of the architecture and learning method of AIVE might explain the higher estimated reward of AIVE’s policy to those of clinicians. First, neural network architecture, which was used to build AIVE, can continuously process the complex relationship between the patient’s status and optimal action, including the dose of anesthetic drugs, hemodynamic or respiratory status, or other features at every second^12^. The actual clinician’s practice might be suboptimal to interpreting tremendous data from various monitoring devices in real-time. Second, the reinforcement learning algorithm helps AIVE to find an optimal policy from our historical data to maximize a cumulative reward^13^. AIVE decides the action for the current patient status considering the future cardiorespiratory changes until complete recovery from general anesthesia. The reinforcement learning model trained by real-world clinical data makes the model find the best policy efficiently.

In the external validation, the estimated reward of AIVE was even higher than those in the internal testing dataset. This may be explained by the differences between the internal and external datasets, as the external dataset included more cases with shorter duration of anesthesia and younger aged patients than the internal dataset. These differences among the datasets may have influenced the discrepancy between the AIVE’s and clinician’s policies. The small number of cases in the external validation dataset may also have affected the relationship between the discrepancy and some variables (SpO_2_), which were not definite.

To the best of our knowledge, this is the first study to develop and validate the reinforcement learning model to suggest the optimal timing of controlling ventilation during anesthesia emergence in surgical patients. Previous studies have developed offline reinforcement learning models to solve complicated medical problems^11,14–17^. One study developed a reinforcement learning model to recommend various interventions, such as administering intravenous fluid and medications, to treat patients with sepsis in the intensive care unit (ICU)^14^. Prasad et al.^15^ reported using a reinforcement learning model for weaning from mechanical ventilation using fitted-Q iteration and the Medical Information Mart for Intensive Care (MIMIC)-III database. Another study developed an inverse reinforcement learning model for discontinuing mechanical ventilation and sedative dosing in critically ill patients^16^. A reward function that can be inferred by inverse reinforcement learning was designed in this study. A recent study developed a reinforcement learning model to suggest an optimized regimen using data from the MIMIC-III database, including tidal volume, a fraction of inspired oxygen (F_I_O_2_), and positive end-expiratory pressure (PEEP). It externally validated the model using another open ICU dataset^11^. Another reinforcement learning model for guiding adequate electrolyte replacement was developed using electronic health records^17^.

The strength of this study is that the reinforcement learning model was developed based on real-world data from actual clinical practice, consisting of high-resolution intraoperative biosignals. Therefore, the reinforcement learning model would better reflect the clinical situation than a model based on a well-refined open dataset. In addition, intraoperative biosignals were obtained from various monitoring devices generally used in the operating room, providing the possibility of application in different clinical environments. The reinforcement learning agent learned the optimal policy using only information about the cardiorespiratory status of the patient during emergence from general anesthesia rather than clinical information. The data used for model development did not require assessment or judgment by clinicians and could be obtained from most hospitals. The reinforcement learning model may develop into a fully automated data-driven clinical decision support system and may facilitate an individualized strategy for controlling ventilation during the emergence from general anesthesia in surgical patients.

Despite these strengths, some important considerations must be taken when deploying our offline reinforcement learning model to real-time settings^18^. First, although the AIVE was designed to propose actions based on biosignals every second, there can be delays in monitoring parameters, communications, and delivering actions. Therefore, comprehensive real-time simulations should be conducted before clinical implementation to ensure the AIVE’s stability. Second, although the AIVE was validated in an external dataset, it was developed using a dataset from a single center that could potentially lead to distributional shifts when deployed in different settings. These shifts could result in the model suggesting suboptimal decisions. Considering this potential instability and biases is crucial before running our model in real-world clinical settings.

This study has some limitations. First, bias associated with the retrospective nature of the study would have affected the results. Second, we excluded cardiac and pediatric patients, as well as patients who underwent thoracic surgery requiring one-lung ventilation; therefore, the model’s performance cannot be generalized to these populations. Third, although we externally validated the model’s performance using a different dataset from an independent hospital, the sample size of the external validation dataset may be relatively small compared with that of the derivation dataset. Therefore, our results must be interpreted cautiously. However, despite the relatively small external validation dataset, the reinforcement learning model policy performed better than the clinicians’ policy in the external validation dataset and in our hospital data. Fourth, our study focused on immediate clinical outcomes in the operating room and PACU. Future research should explore the model’s benefits for long-term and relevant outcomes like delayed emergence and emergence delirium. Fifth, we did not specifically record the precise expertise level of attending anesthesiologists and trainee grades for extubation due to the retrospective nature of the study. However, all extubation processes were performed either by attending anesthesiologists or trainees under the direct supervision of attending anesthesiologists. Due to the retrospective nature of the study, clinical care for the emergence and after emergence cannot be strictly controlled, which might have caused some biases. Sixth, patient comorbidity data was not collected in this study, and there were some missing values, such as arterial blood gas analysis and chest X-ray results, which limited the evaluation of its impact on our results. Future studies should address this aspect to provide clarity. Seventh, the start of anesthesia emergence was defined as when the actual F_I_O_2_ exceeded 70% for automatic detection. However, this led to the exclusion of about 4.7% of patients, introducing potential biases. Eighth, only patients who received volume-controlled ventilation were included in our study. Excluding pressure-controlled or pressure-support ventilation may limit the model’s generalizability. Last, we confined the emergence duration to between 2 and 20 min, excluding 3.9% of patients, which could introduce bias. Future studies may consider employing recent reinforcement learning models capable of stable training with either shorter or longer trajectories.

In conclusion, we developed and validated a reinforcement learning model for the optimal timing of controlling ventilation using intraoperative biosignals during emergence from general anesthesia in surgical patients. A significant discrepancy between the policies of reinforcement learning and clinicians’ policies was associated with greater cardiorespiratory instability, indicating that the reinforcement learning model may have the potential to act as a clinical decision-making support tool. Future prospective validation studies are warranted to confirm our results in the prospective study.

## Methods

### Study design

All data for model development was retrieved from the prospective registry containing the vital signs of surgical patients at the Seoul National University Hospital (SNUH). This prospective registry was approved by the Institutional Review Board (IRB) of SNUH (Approval number: 1408-101-605) and registered at ClinicalTrials.gov (NCT02914444). The IRB also approved the retrospective analysis of the data from this prospective registry (Approval number: 2205-061-1322). The IRB approved the data extraction and analysis for external validation at Seoul National University Bundang Hospital (SNUBH, Approval number: 2207-768-405). The IRBs waived the requirement of written informed consent due to the retrospective nature of this study and the anonymity of the data.

### Data collection

From the registry data, all general anesthesia cases from the derivation cohort (SNUH) between August 2016 and November 2019 were included for model development and internal validation. Cases from the external validation cohort (SNUBH) were included for external validation between January 2022 and June 2022. Additionally, it is worth noting that the majority of cases involving general anesthesia were administered by attending anesthesiologists with several years of experience, and trainees were supervised throughout the process. Cases with the following features were excluded: (1) patient age <18 years, (2) cases in which pressure-controlled ventilation was used, (3) procedures that were not performed under general anesthesia, (4) cases in which the laryngeal mask airway was used rather than an endotracheal tube, (5) cases in which one-lung ventilation was performed using a double-lumen tube, (6) cases that had no tracks regarding critical input variables in the intraoperative biosignals data, (7) cases in which tracheal extubation was not performed at the end of surgery in the operating room, (8) cases in which F_I_O_2_ was not increased before the patient recovered spontaneous breathing, (9) cases in which the duration of emergence was less than 2 min or greater than 20 min, and (10) cases that had no tracks to evaluate the primary outcome.

The intraoperative biosignal data used in the study were collected by a free biosignal collection program (Vital Recorder, ver.1.9.9, accessible at https://vitaldb.net, Seoul, Republic of Korea)^19^. SpO_2_, HR, and SBP were measured using a patient monitor (Solar^TM^ 8000 M, GE Healthcare, Wauwatosa, WI, USA). The indices related to the processed electroencephalogram, such as the bispectral index, electromyogram, and spectral edge frequency, were collected using the brain monitor (BIS Vista^TM^, Medtronic, Dublin, Ireland). In addition, data regarding mechanical ventilation, such as AWP, E_T_CO_2_ level, respiratory compliance, anesthetic agents, PIP, RR, PEEP, and tidal volume, were collected from the anesthesia ventilators (Primus, Dräger, Lübeck, Germany). Among the variables of mechanical ventilation, waveform data, including AWP and E_T_CO_2_ from the anesthesia ventilators, were sampled at a rate of 62.5 Hz. In comparison, other variables were sampled at a rate of 0.14 Hz. cardiorespiratory-related variables from the patient monitor were sampled at 2 Hz. For handling these time-varying variables, we up-sampled using linear interpolation followed by forward and backward filling methods or down-sampled to bring all variables to 10 Hz. We adopted the maximum value with a one-second time window for our model development.

### Anesthesia management

The patients received balanced anesthesia using sevoflurane inhalation and a target-controlled remifentanil infusion or total intravenous anesthesia. For those who received balanced anesthesia, propofol was used for anesthesia induction with a bolus dose of 1.0–2.0 mg/kg, and anesthesia was maintained with sevoflurane and the effect-site target-controlled infusion of remifentanil. The sevoflurane concentration was usually maintained as 0.6–0.8 minimum alveolar concentration, while the target-controlled infusion of remifentanil was usually maintained as 1–4 ng/ml based on hemodynamic changes. In cases of total intravenous anesthesia, target-controlled infusions of propofol and remifentanil were used. Propofol concentrations were usually adjusted to maintain the bispectral index of 40–60, and remifentanil was maintained at 1–4 ng/ml based on hemodynamic changes. An infusion pump (Orchestra^®^, Base Primea with module DPS, Fresenius Kabi AG, Bad Homburg, Germany) was used for target-controlled infusion of remifentanil or propofol. At the end of the surgery, any anesthetics were discontinued, and anesthesia emergence and extubation were performed at the discretion of attending anesthesiologists after administering a reversal agent of the neuromuscular blocking agent. According to the institution’s policy, the emergence process was carried out by attending anesthesiologists or trainees under the direct supervision of attending anesthesiologists.

### Outcome measurements

The primary outcome of the study was the time duration (in seconds) of cardiorespiratory instability during anesthesia emergence, which was defined by a composite outcome based on a combination of the following parameters: SpO_2_, HR, and SBP. The duration of cardiorespiratory instability was quantified by measuring the combined time duration during which any of the following parameters exceeded predefined thresholds: SpO_2_ below 95%, or HR or SBP showing changes greater than 20% changes from their baseline values. The secondary outcomes included the time duration of each following variable: SpO_2_ (<95%), HR (>20% changes from the baseline), SBP (>20% changes from the baseline), PIP (>20% changes from the baseline), and apnea time (E_T_CO_2_ <2 mmHg). We additionally included the following postoperative outcomes as secondary outcomes: cardiorespiratory parameters (SBP, HR, RR, and SpO_2_), clinical outcomes (the length of hospital stay, length of PACU stay, and postoperative 30-day in-hospital mortality), arterial blood gas analysis (partial pressures of oxygen [PaO_2_] and carbon dioxide [PaCO_2_]), and chest X-ray results within 48 h after surgery. The specific threshold values for each parameter are presented in Supplementary Table 1.

The SHAP method, which is based on game theory and provides importance scores for each feature, has been used in the medical research field to present the interpretability of model^20^. Our study also used this method to present how each feature in the state space was attributed to each policy, with 500 weak learners applied in the internal test dataset.

### Markov decision process

The problem regarding optimal ventilation control during anesthesia emergence can be formulated as a Markov decision process (MDP), with state space $S\subseteq {{\mathbb{R}}}^{n}$, where collected features $S$ and action space $A\,\in \,{\mathbb{R}}$ include mechanical or manual ventilation on (${a}^{{{\mathrm{vent}}}{{\mathrm{on}}}}:=1$) or off (${a}^{{{\mathrm{vent}}}{{\mathrm{off}}}}:=0$). The reward $R:S\times A{\mathbb{\to }}{\mathbb{R}}$ depends on the current 2-tuple of state and action. Therefore, given a state $s\in S$, the policy is defined as a probability distribution over the action space $A$, $\pi \left(\cdot\, |s\right)\in \varDelta A$, where $\rho \in \varDelta S$ is the distribution of the initial state, ${s}_{0}$. The probability of a $T$-step trajectory-making transition matrix is defined as follows:

1 $$P(\tau {\rm{|}}{\pi }_{\theta })\triangleq \rho ({s}_{0})\mathop{\prod }\limits_{t=0}^{T-1}P({s}_{t+1}{\rm{|}}{s}_{t},{a}_{t}){\pi }_{\theta }\left({a}_{t}\right|{s}_{t})$$

With the discounting factor, $\gamma$ $\in \,[0,1)$ for future rewards, our ventilation decision problem can be formulated into MDP to create the following value function:

2 $${V}_{\pi }\left(s\right)\,=\,{\mathbb{E}}\left[{R}_{t}{{|}}{s}_{0}=s\right]{\mathbb{=}}{\mathbb{E}}\left[\mathop{\sum }\limits_{t=0}^{T-1}{\gamma }^{t}{r}_{t}{\rm{|}}{s}_{0}=s\right]$$

Moreover, the action-value function (known as the Q-function), ${Q}_{\pi }:S\times A\to R$, can be defined as follows:

3 $${Q}_{\pi }\left(s,\,a\right)\,=\,{\mathbb{E}}\left[{R}_{t}{{|}}({s}_{0},\,{a}_{0})=(s,\,a)\right]\,=\,{\mathbb{E}}\left[\mathop{\sum }\limits_{t=0}^{T-1}{\gamma }^{t}{r}_{t}{{|}}({s}_{0},\,{a}_{0})=(s,\,a)\right]$$

### Reinforcement learning model

Offline reinforcement learning has emerged as an alternative to the typical online setting for reinforcement learning algorithms, as it can use a fully fixed dataset of trajectories without any further interactions with the environment^21^. This offline setting is suitable for the medical field as it enables using existing datasets made by clinicians’ decision-making for real-world patients. Moreover, offline reinforcement learning does not pose any risk to the patients. However, recent studies have shown that conventional reinforcement learning algorithms yield poor performance in offline settings due to extrapolation errors where the values are estimated from state-action pairs and are not included in the existing dataset^22,23^. Therefore, we adopted conservative-Q learning, which learns conservative-Q-function such that the expected value of a policy under this Q-function lower-bounds its true value to reduce overestimation in out-of-distribution actions^24^. This algorithm has yielded better performance than conventional reinforcement learning algorithms. It has been applied in a few medical tasks, including optimizing mechanical ventilation control or sepsis treatment strategy for intensive care unit patients^25,26^. Each definition used in our reinforcement learning model has been described in the following sections.

*T*-step trajectory was defined by the emergence duration as the time from the beginning of waking patients from general anesthesia to the end of the E_T_CO_2_ monitoring. The start of emergence was defined as increasing F_I_O_2_ to 70% or higher. The minimum and maximum bounds of the length of *T*-step trajectory were determined, considering both the training stability of the reinforcement learning model and the usual length of the anesthesia recovery. We categorized the patients’ ventilation status (ventilation-dependent or ventilation-independent) and extubation status (intubated or extubated status) into two states. The ventilation-dependent status indicates that the lungs were mechanically ventilated by the anesthesia ventilator or manually ventilated by clinicians. In contrast, the ventilation-independent status indicates that the patient had spontaneous breathing, not requiring ventilation support by the anesthesia ventilator or clinicians. Lastly, intubated or extubated status represented whether the endotracheal tube was present in the trachea.

The state space consists of the following 10 features at time *t*: effect-site concentrations of propofol and remifentanil; end-expiratory pressure of sevoflurane; PIP; tidal volume, the moving averaged AWP and E_T_CO_2_ within 6 s; HR; SpO_2_; SBP; the presence of spontaneous breathing; the cumulation of apnea time, defined as E_T_CO_2_ <2 mmHg, after turning the mechanical ventilator off; and current ventilation and extubation status (ventilation-dependent/independent and intubated/extubated status). The presence of spontaneous breathing was detected through abrupt changes in airway pressure. These changes indicate sudden increases (at least 5 cmH_2_O higher than the previous maximum AWP within 15–30 s) or sudden decreases in AWP (at least 3 cmH_2_O lower than the PEEP setting) due to the patient-ventilator asynchrony by spontaneous breathing. The earliest time point that meets the criteria was defined as the moment when spontaneous breathing returns. Furthermore, apnea time was cumulated while E_T_CO_2_ was lower than 2 mmHg. The action variable was selected at each time $t$ from two discrete action candidates, including ventilation (*a*^vent on^) or non-ventilation (*a*^vent off^).

### Reward function

The AIVE maintained cardiorespiratory stability, adequate oxygenation, and decarboxylation during emergence from general anesthesia. Therefore, we divided the reward system into two parts as ${r}_{{t}}$ was defined by the penalties from cardiorespiratory parameters (${r}^{{{\mathrm{CR}}}}$) in the next time step. Specifically, ${r}^{{{\mathrm{CR}}}}$ consists of oxygen saturation below 97% $({v}^{{{\mathrm{SPO}}}{\mathrm{2}}})$, cumulated apnea time over 6 s $({v}^{{{\mathrm{apnea}}}})$, HR $({v}^{{{\mathrm{HR}}}})$, SBP $({v}^{{{\mathrm{SBP}}}})$, and PIP $({v}^{{{\mathrm{PI}}}{\mathrm{P}}})$ showing a 20% increase from the baseline condition defined by the averaged values for 10 s when F_I_O_2_ begins to rise. Lastly, the reward system was balanced using four constants (${{\rm{\alpha }}}_{k},k\in \{\mathrm{1,2,3,4}\})$ through anesthesiology experts’ knowledge, and the lower bound of *r*_*t*_ was set at −20, which was the first quantile of elements in reward space, as shown below:

4 $${r}_{t}={\rm{max }}(-20,-{r}_{t}^{{{{\mathrm{CR}}}}})$$

5 $${r}_{t}^{{{\mathrm{CR}}}}\left(s,a\right)\,{{:= }}\,{v}^{{{\mathrm{apnea}}}}+{\alpha }_{1}\cdot {v}^{{{\mathrm{SPO}}}{\mathrm{2}}}+{\alpha }_{2}\cdot {v}^{{{\mathrm{HR}}}}+{\alpha }_{3}\cdot {v}^{{{\mathrm{SBP}}}}+{\alpha }_{4}\cdot {v}^{{{\mathrm{PIP}}}}{{\mathrm{for}}}\,{{\mathrm{all}}}\,\left(s,a\right)\in S\times A$$

### Performance evaluation

To compare the performance of the AIVE’s policy with that of the clinicians’ policy, we adopted the fitted-Q-evaluation (FQE) method with bootstrapping to provide the confidence interval for each policy among 300 different models^27,28^. The derivation cohort dataset was randomly divided into two datasets (training set [85%] and testing set [15%]) for each model. Three hundred models were built via various random splits (82.3%) of the training dataset and evaluated by the remaining validation set (17.7%); each model’s whole learning scheme was consistent. All training was conducted on an NVIDIA RTX A6000 GPU.

Using the FQE method, we compared the 95% lower bound of the AIVE’s performance return with the 95% upper bound of clinicians’ rewards to evaluate our new policy conservatively, as suggested by previous RL studies^11,14,29^. Finally, the model which maximized the 95% lower bound of the AIVE’s policy was selected for further outcome measurement.

### Statistical analysis

Python 3.8.0 (Python Software Foundation, Wilmington, DE, USA) was used for signal preprocessing, model development and validation, statistical testing, and visualization. Statistical analyses of primary and secondary outcomes were conducted using Kendall’s rank correlation for continuous outcomes and the point-biserial correlation for categorical outcomes. All statistics for continuous variables were reported with point estimates and 95% confidence intervals, and those for categorical variables were reported with counts (frequencies) or proportions. The original significance level was set at 0.05. The Bonferroni correction was utilized to account for multiple comparisons, considering one primary and 24 secondary outcomes. Therefore, a *P*-value <0.002 was considered statistically significant.

### Reporting summary

Further information on research design is available in the Nature Research Reporting Summary linked to this article.

### Supplementary information

Supplementary Information

Reporting Summary

## Data Availability

The public dataset to run the code for this study is available at https://vitaldb.net/. The data supporting this study’s findings are also available from the corresponding author upon reasonable request.

## Code availability

The code to generate the result of this study can be accessed at https://github.com/HyeonhoonLee/AIVE.
