# Supplementary material for: Development and validation of a reinforcement learning model for ventilation control during emergence from general anesthesia
Source: NPJ Digit Med. 2023 Aug 14;6:145. doi: 10.1038/s41746-023-00893-w (PMC10425339; doi:10.1038/s41746-023-00893-w)
Supplement: Supplementary file 1 — Supplementary Information [file 41746_2023_893_MOESM1_ESM.pdf]

Supplementary Figures 1. The changes in the secondary outcomes depend on the degree of time discrepancy between the AIVE's and clinicians' policies in the internal test set. The plots were produced with 3,000 resamplings, and the shaded area represents the 95% confidence interval. SpO2, peripheral oxygen saturation; SBP; systolic blood pressure; HR, heart rate; PIP, peak inspiratory pressure; AIVE, Artificial Intelligence model for Ventilation control during Emergence.

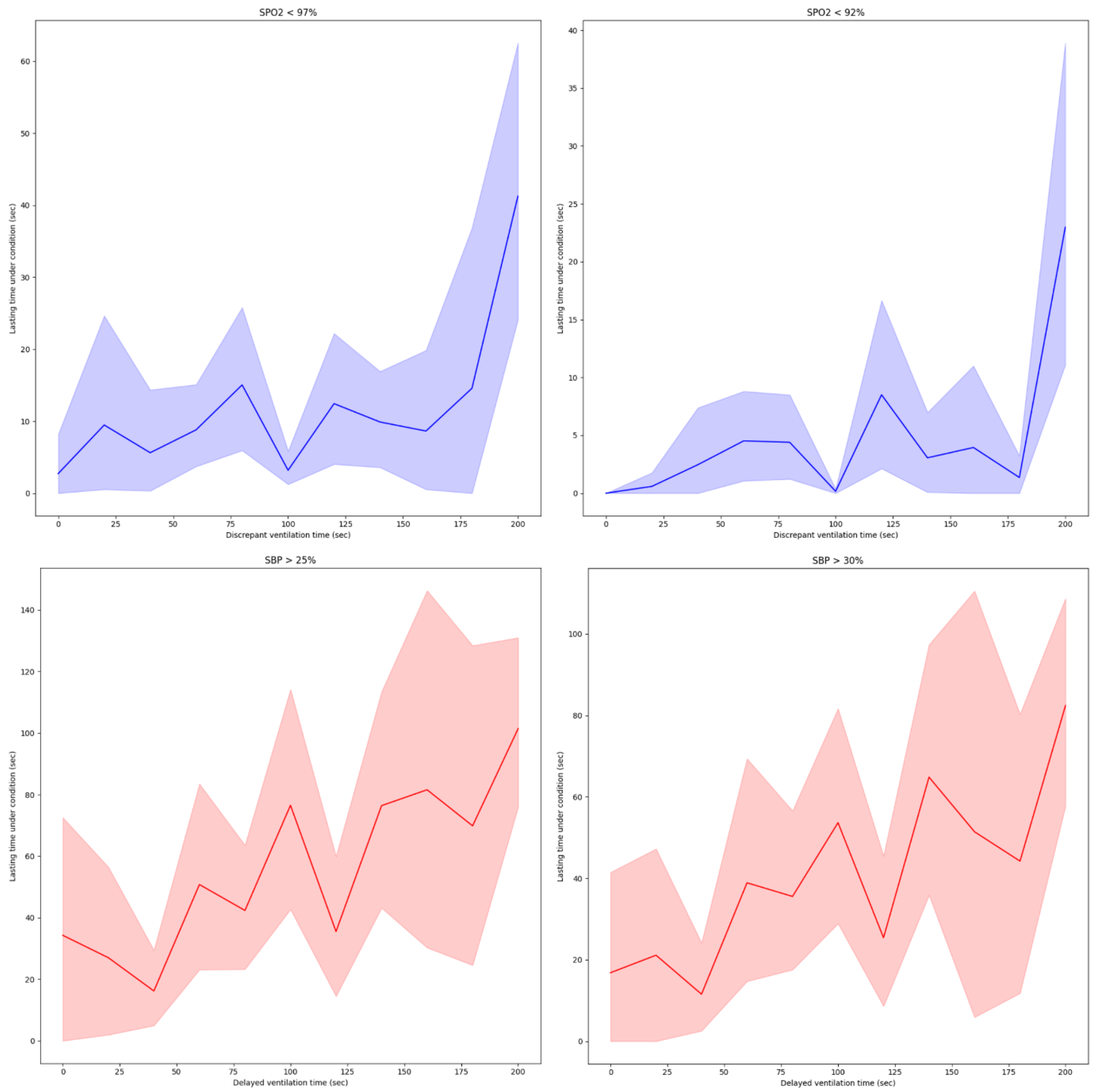

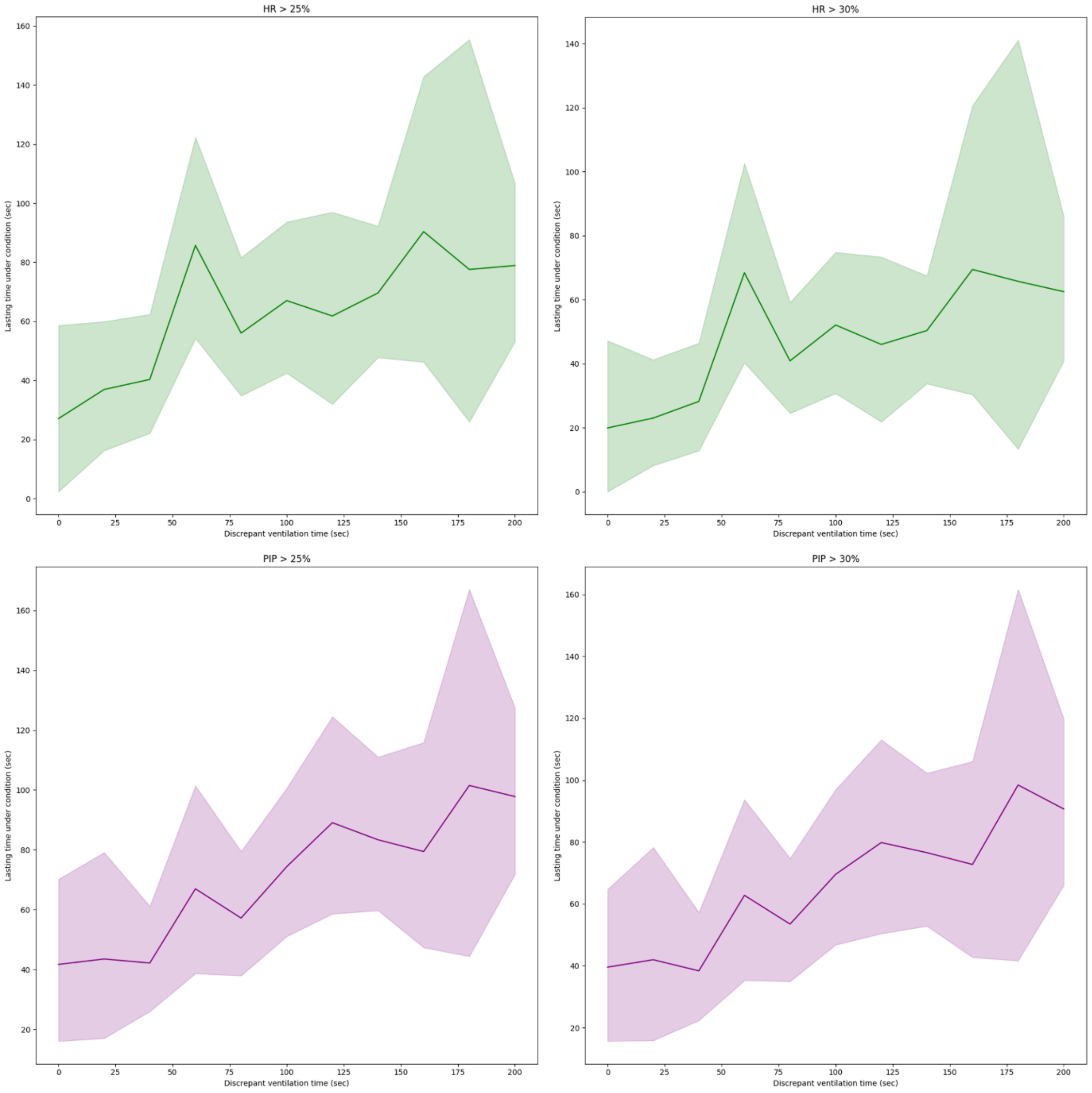
HR > 25%
HR > 30%
PIP > 25%
PIP > 30%
Lasting time under condition (sec)
Discrepant ventilation time (sec)

Supplementary Figures 2. The changes in the secondary outcomes depend on the degree of time discrepancy between the AIVE's and clinicians' policies in the external test set. The plots were produced with 3,000 resamplings, and the shaded area represents the 95% confidence interval. SpO2, peripheral oxygen saturation; SBP; systolic blood pressure; HR, heart rate; PIP, peak inspiratory pressure; AIVE, Artificial Intelligence model for Ventilation control during Emergence.

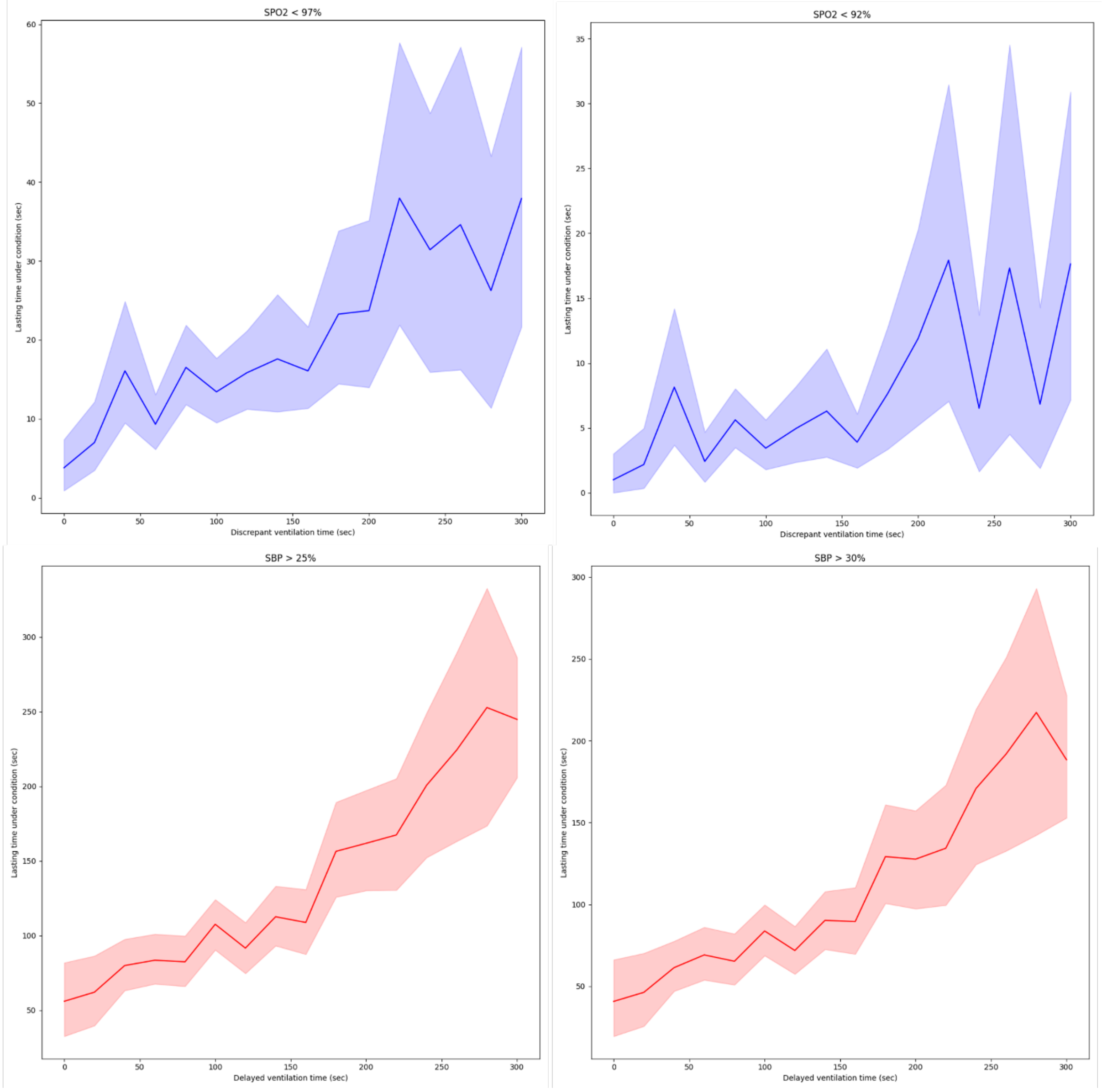

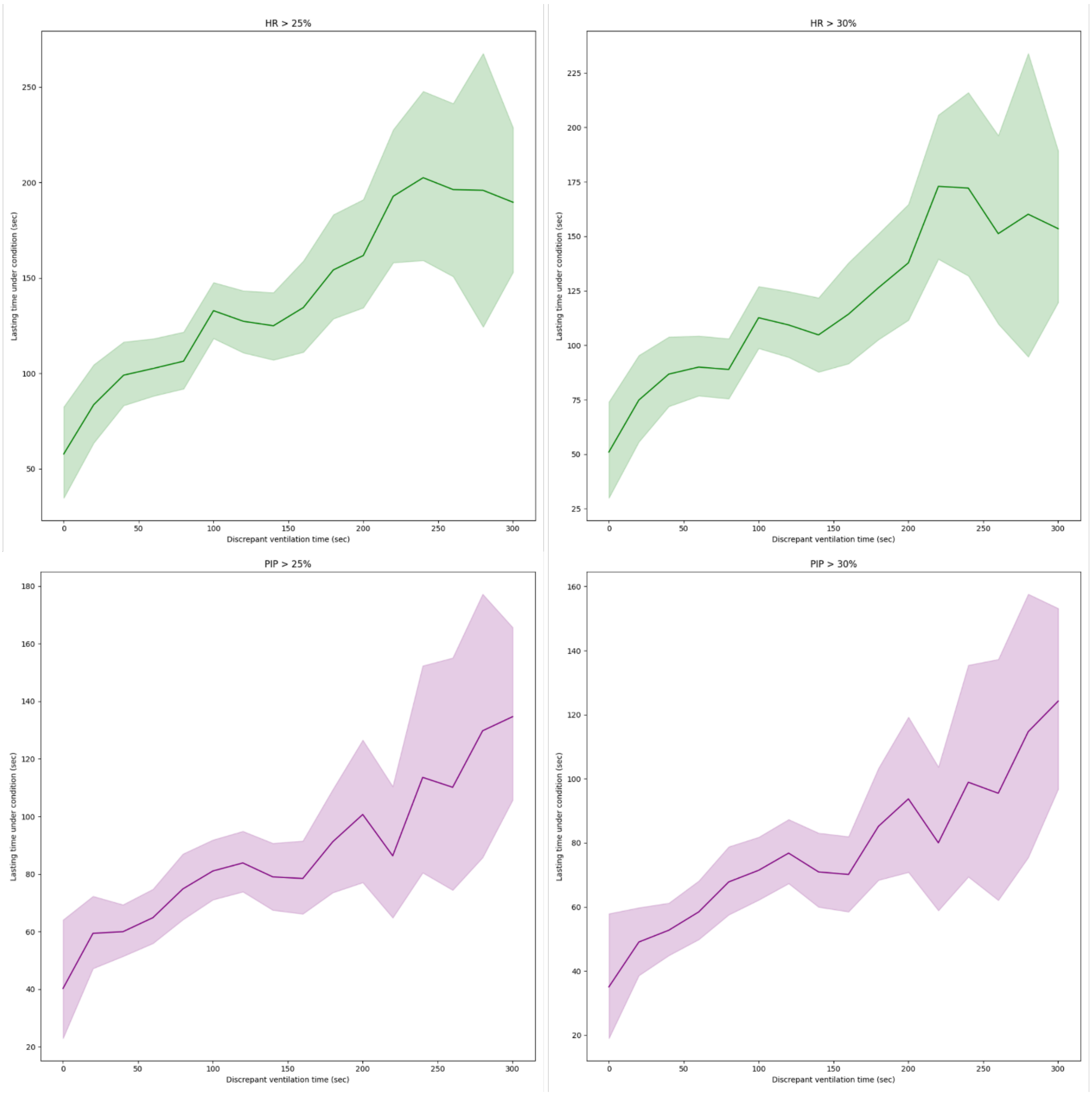
HR > 25%
HR > 30%
PIP > 25%
PIP > 30%
Lasting time under condition (sec)
Discrepant ventilation time (sec)

Supplementary Table 1. The parameters and their thresholds for outcome measurement

Variables	Threshold	Units
Primary outcome (SpO_2/HR/SBP)	95/20/20	%
Secondary outcome		
SpO_2	97, 95, 92	%
HR	20, 25, 30	%
SBP	20, 25, 30	%
PIP	20, 25, 30	%
E_TCO_2 (Apnea)	2	mmHg

SpO_2, peripheral oxygen saturation; HR, heart rate; SBP, systolic blood pressure; PIP, peak inspiratory pressure; E_TCO_2, end-tidal carbon dioxide concentration.

Supplementary Table 2. The correlation between the policy discrepancy and the intraoperative cardiorespiratory parameters (secondary outcome) in the internal and external test set

	Internet test set			External test set		
	N	Coefficient	*P*-value	N	Coefficient	*P*-value
Intraoperative cardiorespiratory parameters						
SpO_2 < 97%	0.102	0.102	<0.001	0.134	0.134	<0.001
SpO_2 < 92%	0.076	0.076	<0.001	0.134	0.134	<0.001
HR > 25%	0.124	0.124	<0.001	0.027	0.027	0.454
HR > 30%	0.093	0.093	<0.001	0.039	0.039	0.277
SBP > 25%	0.184	0.184	<0.001	0.186	0.186	<0.001
SBP > 30%	0.173	0.173	<0.001	0.179	0.179	<0.001
PIP > 25%	0.075	0.075	<0.001	0.109	0.109	0.001
PIP > 30%	0.073	0.073	<0.001	0.100	0.100	0.003

Kendall's rank correlation analysis was performed. SpO2, peripheral oxygen saturation; HR, heart rate; SBP; systolic blood pressure; PIP, peak inspiratory pressure.

Supplementary Table 3. Subgroup analysis for the correlation between the policy discrepancy and the length of hospital stay in the internal and external test set

	Internet test set			External test set		
	N	Coefficient	*P*-value	N	Coefficient	*P*-value
Age						
>50	1481	0.086	<0.001	240	0.040	0.377
<=50	665	0.091	<0.001	156	-0.037	0.547
Sex						
Female	1091	0.086	<0.001	221	0.018	0.722
Male	1055	0.096	<0.001	175	-0.052	0.332
Surgery type						
General surgery	1378	0.085	<0.001	340	0.027	0.481
Urology surgery	239	0.051	0.250	1	NA	
Orthopedic surgery	161	0.149	0.007	8	-0.077	0.797
Gynecological surgery	114	0.155	0.028	40	0.092	0.471
Neurosurgery	93	0.092	0.207	6	-0.552	0.126
Plastic surgery	92	0.054	0.477	0	NA	
Thoracic surgery	19	0.071	0.674	0	NA	
Others	20	0.073	<0.001	1	NA	

Kendall's rank correlation analysis was performed. NA, not available.
