# Supplementary material for: Development and validation of a reinforcement learning model for ventilation control during emergence from general anesthesia
Source: NPJ Digit Med. 2023 Aug 14;6:145. doi: 10.1038/s41746-023-00893-w (PMC10425339; doi:10.1038/s41746-023-00893-w)
Supplement: Supplementary file 2 — Reporting Summary [file 41746_2023_893_MOESM2_ESM.pdf]

nature portfolio

Corresponding author(s): Hyung-Chul Lee

Last updated by author(s): May 23, 2023

Reporting Summary

Nature Portfolio wishes to improve the reproducibility of the work that we publish. This form provides structure for consistency and transparency in reporting. For further information on Nature Portfolio policies, see our Editorial Policies and the Editorial Policy Checklist.

Statistics

For all statistical analyses, confirm that the following items are present in the figure legend, table legend, main text, or Methods section.

n/a	Confirmed	
☐	☒	The exact sample size (*n*) for each experimental group/condition, given as a discrete number and unit of measurement
☐	☒	A statement on whether measurements were taken from distinct samples or whether the same sample was measured repeatedly
☐	☒	The statistical test(s) used AND whether they are one- or two-sided<br>*Only common tests should be described solely by name; describe more complex techniques in the Methods section.*
☐	☒	A description of all covariates tested
☐	☒	A description of any assumptions or corrections, such as tests of normality and adjustment for multiple comparisons
☐	☒	A full description of the statistical parameters including central tendency (e.g. means) or other basic estimates (e.g. regression coefficient) AND variation (e.g. standard deviation) or associated estimates of uncertainty (e.g. confidence intervals)
☐	☒	For null hypothesis testing, the test statistic (e.g. F, t, r) with confidence intervals, effect sizes, degrees of freedom and P value noted<br>*Give P values as exact values whenever suitable.*
☐	☒	For Bayesian analysis, information on the choice of priors and Markov chain Monte Carlo settings
☐	☒	For hierarchical and complex designs, identification of the appropriate level for tests and full reporting of outcomes
☐	☒	Estimates of effect sizes (e.g. Cohen's *d*, Pearson's *r*), indicating how they were calculated

Our web collection on statistics for biologists contains articles on many of the points above.

Software and code

Policy information about availability of computer code

Data collection	The Vital Recorder (available at https://doi.org/10.1038/s41598-018-20062-4) and the clinical data warehouse system of the hospitals
Data analysis	A custom-developed program written in Python 3.8.0 (Python Software Foundation, Wilmington, DE, USA) was used for signal preprocessing, model development and validation, statistical testing, and visualization.

For manuscripts utilizing custom algorithms or software that are central to the research but not yet described in published literature, software must be made available to editors and reviewers. We strongly encourage code deposition in a community repository (e.g. GitHub). See the Nature Portfolio guidelines for submitting code & software for further information.

Data

Policy information about availability of data

All manuscripts must include a data availability statement. This statement should provide the following information, where applicable:

- Accession codes, unique identifiers, or web links for publicly available datasets
- A description of any restrictions on data availability
- For clinical datasets or third party data, please ensure that the statement adheres to our policy

The dataset used in this study is not publicly available. However, the data of this study can be provided if there is a reasonable request to the corresponding author.

Research involving human participants, their data, or biological material

Policy information about studies with human participants or human data. See also policy information about sex, gender (identity/presentation), and sexual orientation and race, ethnicity and racism.

Reporting on sex and gender	We used the term sex indicating the biological attribute. The values were extracted from the medical records in the clinical data warehouse.
Reporting on race, ethnicity, or other socially relevant groupings	Asian race is mostly studied in this study. We didn't used the race as a proxy for socioeconomic status.
Population characteristics	Age, sex, height, weight, total duration of anesthesia (min), duration of anesthesia emergence (sec), Surgery type
Recruitment	People who underwent surgery during the study periods were included
Ethics oversight	This study was approved by the institutional review boards of the hospitals.

Note that full information on the approval of the study protocol must also be provided in the manuscript.

Field-specific reporting

Please select the one below that is the best fit for your research. If you are not sure, read the appropriate sections before making your selection.

☒ Life sciences ☐ Behavioural & social sciences ☐ Ecological, evolutionary & environmental sciences

For a reference copy of the document with all sections, see nature.com/documents/nr-reporting-summary-flat.pdf

Life sciences study design

All studies must disclose on these points even when the disclosure is negative.

Sample size	Sample size was determined by the number of surgical cases during the study periods.
Data exclusions	1) patient age < 18 years, 2) cases in which pressure-controlled ventilation was used, 3) procedures that were not performed under general anesthesia, 4) cases in which the laryngeal mask airway was used rather than an endotracheal tube, 5) cases in which one-lung ventilation was performed using a double-lumen tube, 6) cases that had no tracks regarding critical input variables in the intraoperative biosignals data, 7) cases in which tracheal extubation was not performed at the end of surgery in the operating room, 8) cases in which FiO2 was not increased before the patient recovered spontaneous breathing, 9) cases in which the duration of emergence was less than 2 min or greater than 20 min, and 10) cases that had no tracks to evaluate the primary outcome
Replication	The public dataset to run the code for this study is available at https://vitaldb.net/. The data supporting this study's findings are also available from the corresponding author upon reasonable request.
Randomization	No randomization was performed in this study.
Blinding	No blinding was performed in this study.

Reporting for specific materials, systems and methods

We require information from authors about some types of materials, experimental systems and methods used in many studies. Here, indicate whether each material, system or method listed is relevant to your study. If you are not sure if a list item applies to your research, read the appropriate section before selecting a response.

Materials & experimental systems

n/a	Involved in the study	
☒	☐	Antibodies
☒	☐	Eukaryotic cell lines
☒	☐	Palaeontology and archaeology
☒	☐	Animals and other organisms
☒	☐	Clinical data
☒	☐	Dual use research of concern
☒	☐	Plants

Methods

n/a	Involved in the study	
☒	☐	ChIP-seq
☒	☐	Flow cytometry
☒	☐	MRI-based neuroimaging
